# Artificial intelligence-integrated multimodal retinal imaging for early detection and risk stratification of systemic vascular and neurodegenerative diseases

**DOI:** 10.3389/fneur.2026.1885303

**Published:** 2026-08-12

**Authors:** Kaili Du, Yuqiang Zheng, Zhuoshi Wang

**Affiliations:** 1 Eye Genebank, He University, Shenyang, China; 2 He Eye Specialists Hospital, Shenyang, China

**Keywords:** artificial intelligence, diabetic retinopathy, hypertensive retinopathy, multimodal deep learning, neurodegeneration, retinal imaging

## Abstract

**Background:**

We developed and validated RetinalVNG-Net, a multimodal deep learning framework for simultaneous risk stratification of hypertensive retinopathy, diabetic retinopathy, and neurodegenerative-associated retinal changes from integrated fundus photography, optical coherence tomography, and clinical metadata.

**Methods:**

This retrospective multi-center study included 2,740 subjects from three independent ophthalmology centers (January 2019–December 2023). Centers A and B (*n* = 2,220) constituted the development set, with a stratified 15% subset (*n* = 333) reserved for hyperparameter tuning and the remainder (*n* = 1,887) used for five-fold cross-validation. Center C (*n* = 520) served as a geographically distinct, device-heterogeneous external test set. RetinalVNG-Net employs a RETFound ViT-Large fundus encoder, a dual-stream Optical Coherence Tomography (OCT) branch (ResNet-3D-18 for volumetric B-scans and 2D-CNN for layer thickness maps), and a tabular transformer for metadata, fused via cross-modal attention. An auxiliary regression head outputs a continuous Retinal Biological Age Gap (RBAG) score as an interpretable severity biomarker.

**Results:**

Internal cross-validation yielded macro-averaged AUC-ROC 0.957 (±0.008), sensitivity 0.913, specificity 0.941, and F1 0.908 across four classes. On the external test set, macro-averaged AUC-ROC reached 0.944 (95% CI 0.922–0.958), with per-class AUCs of 0.963 (hypertensive retinopathy), 0.951 (diabetic retinopathy), 0.924 (neurodegenerative changes), and 0.938 (controls). RetinalVNG-Net significantly outperformed the best single-modality fundus model (macro-AUC 0.944 vs. 0.901; *p* < 0.001).

**Conclusion:**

RetinalVNG-Net demonstrates promising robustness and generalizability for simultaneous multimodal risk stratification of vascular and neurodegenerative retinal changes across diverse devices and settings, supporting further evaluation as a tool for risk stratification of retinal manifestations associated with systemic vascular and neurodegenerative disease. Prospective longitudinal studies would be required to establish value for early or predictive detection.

## 1 Introduction

Cardiovascular disease and neurodegenerative disorders collectively represent two of the most prevalent and debilitating categories of chronic illness worldwide, together accounting for a disproportionate share of global morbidity, mortality, and healthcare expenditure (1). Hypertensive vascular disease affects more than 1.3 billion adults globally and remains the single largest attributable risk factor for stroke, heart failure, and end-organ damage (2). Diabetic retinopathy, a microvascular complication affecting approximately one-third of individuals with diabetes mellitus, frequently heralds broader systemic vascular compromise before overt cardiovascular events occur (3, 4). Neurodegenerative conditions, including Alzheimer’s disease and Parkinson’s disease, impose an escalating burden on aging populations, with combined global prevalence projected to exceed 150 million by 2050 (5). The clinical challenge shared across these conditions is consistent: by the time symptoms prompt investigation, pathological processes have often progressed beyond the window of greatest therapeutic benefit. Tools that can stratify risk and classify disease-related retinal changes from a single accessible imaging encounter therefore address a clinically important need, and represent a necessary step toward the longer-term goal of earlier, preclinical identification (6).

The retina offers a uniquely accessible and anatomically informative window into both systemic vascular and neurodegenerative pathology. As an embryological extension of the central nervous system, the retina shares vascular autoregulatory mechanisms with the cerebral microcirculation and exposes neural tissue, including retinal ganglion cells and the axons comprising the retinal nerve fiber layer, that undergo measurable degeneration in concert with cortical and subcortical neuronal loss (7, 8). Structural changes in the peripapillary retinal nerve fiber layer (pRNFL) and the macular ganglion cell–inner plexiform layer (mGCIPL) have been documented in Alzheimer’s disease and Parkinson’s disease, often preceding clinical diagnosis by several years (9–11). Simultaneously, microvascular alterations in retinal arteriolar caliber, vessel tortuosity, and fractal dimension reflect systemic hypertensive and diabetic vascular remodeling with high anatomical fidelity (12). Retinal fundus photography and optical coherence tomography (OCT) can capture these structural and vascular signatures non-invasively, rapidly, and at relatively low cost, positioning the eye as a tractable screening interface for systemic disease risk assessment, a paradigm increasingly referred to as oculomics (13).

The emergence of deep learning has substantially advanced the analytical capacity of retinal imaging. Convolutional neural networks and, more recently, vision transformer-based architectures have demonstrated the ability to detect cardiovascular risk factors, predict incident myocardial infarction and heart failure, identify neurodegeneration-associated retinal thinning, and estimate biological age from fundus photographs with performance approaching or exceeding that of conventional clinical risk models (14, 15). The development of RETFound, a large-scale vision transformer pretrained on 1.6 million unlabelled retinal images via self-supervised masked autoencoding, established a foundation model capable of generalizable representation learning across a wide spectrum of ocular and systemic tasks (16). Landmark studies using deep learning applied to retinal photographs have demonstrated cardiovascular risk stratification via coronary artery calcium score prediction, detection of Alzheimer’s disease from fundus images in multicentre cohorts, and prediction of Parkinson’s disease risk through retinal age gap estimation (17, 18). These findings establish proof of concept for retinal imaging (19).

However, three critical limitations constrain the clinical translation of existing approaches. First, the vast majority of published models are unimodal, relying exclusively on color fundus photography without incorporating the complementary structural depth information encoded in OCT volumetric data or the quantitative contextual information available in clinical metadata. Fundus photography captures two-dimensional vascular and surface morphology, whereas OCT provides layer-resolved structural quantification of the neuroretinal architecture, information that is particularly relevant to the detection of early neurodegeneration-associated retinal thinning (20). The exclusive reliance on a single imaging modality therefore forfeits a substantial proportion of the diagnostic signal available in standard ophthalmic examinations (21). Second, existing models are predominantly task-specific, trained to predict a single disease or biomarker in isolation. This design precludes simultaneous risk stratification across multiple disease categories from a single imaging encounter, limiting clinical efficiency and failing to capture the shared pathophysiological substrate, namely, microvascular and neuroretinal deterioration, that underlies both vascular and neurodegenerative conditions (22). Third, the generalizability of reported models across imaging devices from different manufacturers and across independent patient populations remains incompletely characterized, as most studies have been validated in single-center or single-device settings. The heterogeneity of real-world retinal imaging infrastructure, encompassing devices from multiple vendors with distinct acquisition protocols and image characteristics, represents a fundamental challenge to clinical deployment that has rarely been addressed with rigorous multi-device, multi-center external validation.

To address these limitations, we present RetinalVNG-Net (Retinal Vascular-Neuro-Glial Network), a multimodal deep learning framework integrating color fundus photography, OCT volumetric data, and structured clinical metadata for simultaneous risk stratification of hypertensive retinopathy, diabetic retinopathy, and neurodegenerative-associated retinal changes across three independent clinical centers using three distinct imaging device combinations. The architecture employs a cross-attention fusion mechanism to integrate heterogeneous modality representations and introduces the Retinal Biological Age Gap (RBAG) as a continuous, interpretable risk biomarker derived from the auxiliary regression head. RBAG quantifies the discrepancy between model-predicted retinal biological age and chronological age, and is introduced here as an exploratory candidate severity index whose association with disease across the target conditions is examined rather than assumed. The model is rigorously evaluated through stratified five-fold cross-validation on a development cohort and independently validated on a geographically and device-distinct external test set, with comprehensive ablation analysis isolating the contribution of each architectural component. To our knowledge, this is the first study to investigate simultaneous multimodal deep learning-based risk stratification of both systemic vascular and neurodegenerative-associated retinal conditions within a single unified framework, with external validation across multiple imaging platforms.

## 2 Methods

### 2.1 Study design and data collection

This retrospective multi-center study was conducted across three independent ophthalmology centers (Center A, Center B, and Center C), with data collected between January 2019 and December 2023 (Table 1). Centers A and B contributed data for model development and internal validation, while Center C served exclusively as an external test set to evaluate generalizability. The study protocol was approved by the institutional review board (IRB) at each participating center in accordance with the Declaration of Helsinki. Given the retrospective nature of the study and the complete de-identification of all records prior to analysis, a waiver of individual informed consent was granted by each IRB. No identifiable patient information was retained at any stage of data processing. The study protocol was approved by the Institutional Review Board of He Eye Specialists Hospital (Approval No. HESH-2023-018), the Ethics Committee of Shenyang Aier Ophthalmology Hospital (Center A, Approval No. SYAE-2023-042), the Ethics Committee of Shenyang Fourth People’s Hospital (Center B, Approval No. SYSPH-2023-056), and the Institutional Review Board of Dalian Third People’s Hospital (Center C, Approval No. DLTPH-2023-011).

**Table 1:** Summarizes the data collected from each center and their assigned roles in the study.

Variable	Center A	Center B	Center C
Role	Training/Internal Validation	Training/Internal Validation	External Test
Total Subjects (*n*)	1,240	980	520
Fundus Images (*n*)	2,480	1,960	1,040
OCT Volumes (*n*)	2,480	1,960	1,040
Hypertensive Retinopathy (*n*)	410	320	175
Diabetic Retinopathy (*n*)	380	295	160
NRC (*n*)	220	180	95
Healthy Controls (*n*)	230	185	90
Mean Age ± SD (years)	58.3 ± 11.4	56.9 ± 12.1	57.7 ± 11.8
Sex (% Female)	52.1%	49.8%	51.3%
Fundus Camera	Topcon TRC-NW400	Heidelberg Spectralis HRA	Canon CR-2
OCT Device	Topcon Triton DRI-OCT	Zeiss Cirrus HD-OCT 5000	Heidelberg Spectralis OCT

From each center, the following data were collected per patient: color fundus photographs (CFP) of both eyes, OCT B-scans of the macula and optic nerve head, and structured clinical metadata including age, sex, systolic and diastolic blood pressure, fasting blood glucose, HbA1c, serum creatinine, and confirmed clinical diagnosis. Three target conditions were defined. Hypertensive Retinopathy (HR) was diagnosed according to the Keith–Wagener–Barker classification (grade I–IV) by a board-certified ophthalmologist. Diabetic Retinopathy (DR) was graded using the Early Treatment Diabetic Retinopathy Study (ETDRS) severity scale and confirmed by fundus examination. Neurodegenerative-associated Retinal Changes (NRC) constituted the third and most novel target category, defined by the co-occurrence of two mandatory criteria: a confirmed clinical diagnosis of Alzheimer’s disease or Parkinson’s disease by a board-certified neurologist according to established diagnostic criteria (NIA-AA 2018 for Alzheimer’s disease; MDS clinical criteria for Parkinson’s disease), and quantitative retinal thinning on OCT defined as peripapillary retinal nerve fiber layer (pRNFL) thickness below the 5th percentile of age-matched normative values in at least two clock-hour sectors, or macular ganglion cell–inner plexiform layer (mGCIPL) thickness below the 5th percentile normative threshold as determined by the device’s built-in normative database. The dependence between this OCT-based criterion and the OCT model inputs is examined directly through sensitivity analyses reported in Section 3.11. Alzheimer’s and Parkinson’s disease were grouped under a single NRC label because both share a common substrate of neuroretinal degeneration, manifesting as pRNFL and mGCIPL thinning, which constitutes the structural signature the present model is designed to detect; differential discrimination between the two was beyond the scope of this study.

### 2.2 Inclusion and exclusion criteria

A total of 3,481 subjects were initially identified across the three centers as potentially eligible. Of these, 549 were excluded during pre-screening: 201 for age outside the 30–80-year range, 183 for unavailability of both fundus photograph and OCT volume, and 165 for incomplete clinical metadata records. The remaining 2,932 subjects underwent full eligibility assessment.

Inclusion criteria were as follows: age between 30 and 80 years; availability of high-quality fundus photograph and OCT volume for at least one eye; confirmed clinical diagnosis by a board-certified ophthalmologist or neurologist for one of the three target conditions, or documented healthy status confirmed by comprehensive ophthalmic examination; and complete clinical metadata record.

**Figure 1:**
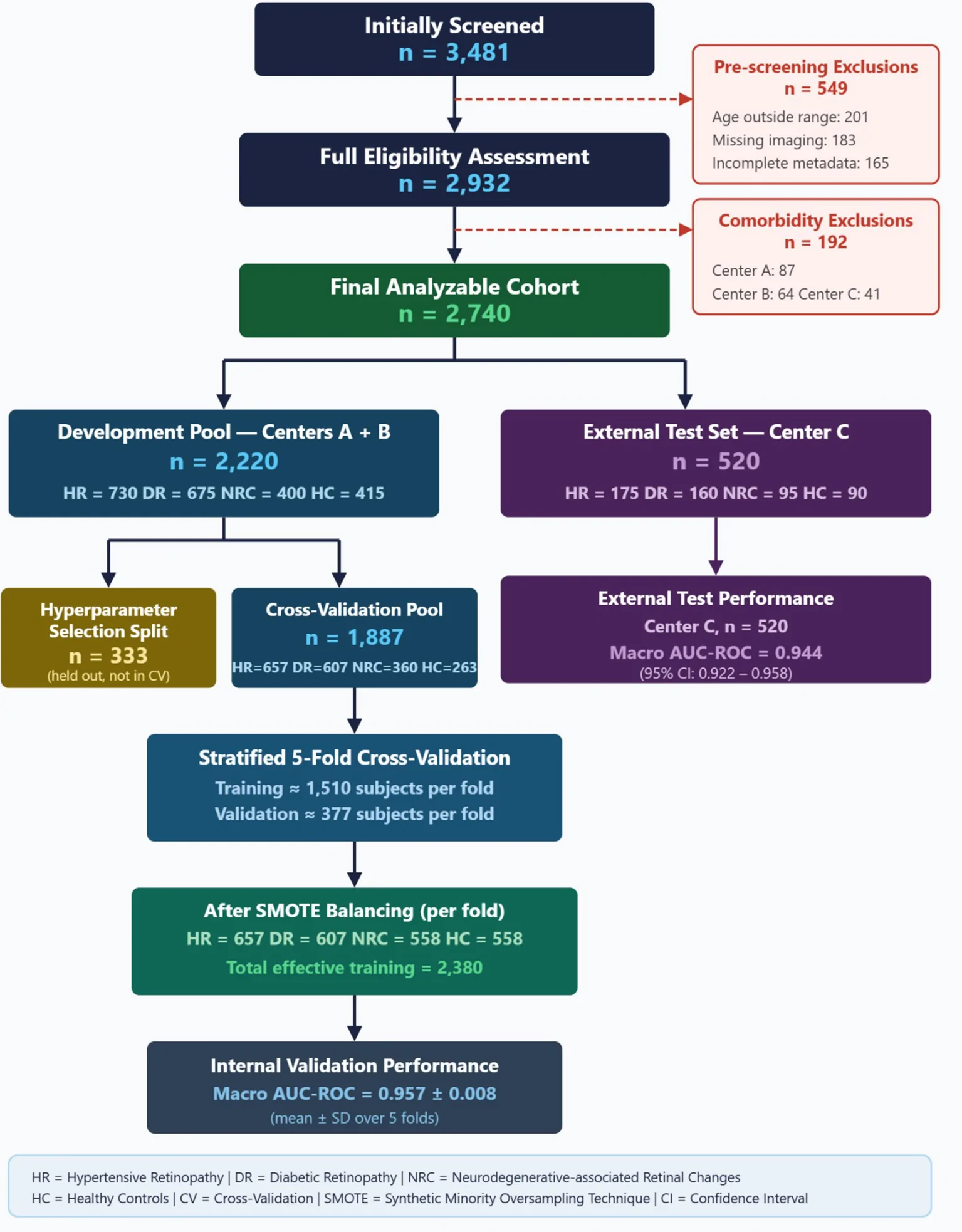
STROBE-compliant enrollment flowchart summarizing subject eligibility and allocation across all three centers.

Exclusion criteria were: presence of media opacity causing image gradability score below 4 on the ETDRS scale; prior intraocular surgery within six months of imaging; coexistence of more than one target condition (to prevent label ambiguity); incomplete OCT volumes with more than 15% missing B-scans; poor-quality fundus images defined by a National Eye Institute gradeability score below 2; and any active inflammatory ocular disease. Following full eligibility assessment, 192 subjects were excluded due to co-occurrence of more than one target condition (Center A = 87, Center B = 64, Center C = 41), yielding a final analyzable cohort of 2,740 subjects. The complete enrollment flow is illustrated in Figure 1.

The flowchart depicts sequential exclusion from initial screening (*n* = 3,481) through pre-screening exclusions (*n* = 549), full eligibility assessment (*n* = 2,932), comorbidity-based exclusions (*n* = 192), and final cohort allocation (*n* = 2,740) into the development pool (Centers A + B, *n* = 2,220) and external test set (Center C, *n* = 520). Within the development pool, the hyperparameter selection split (*n* = 333) and five-fold cross-validation pool (*n* = 1,887) are shown. Per-center class distributions are indicated for each partition.

Image quality assessment was performed independently by two trained graders using a standardized protocol. Inter-rater agreement yielded *κ* = 0.83 (95% CI: 0.79–0.87), indicating strong agreement.

### 2.3 Data splitting and cross-validation strategy

Data from Centers A and B were merged into a development pool of 2,220 subjects. A stratified 15% subset (*n* = 333) was randomly sampled and reserved exclusively for hyperparameter selection. The remaining 1,887 subjects formed the cross-validation pool, divided using stratified five-fold cross-validation preserving proportional class distribution. In each fold, four folds were used for training (~1,510 subjects) and one for internal validation (~377 subjects). Center C (*n* = 520) was designated as the external test set based on three pre-specified criteria: geographic separation, distinct imaging devices, and an independent patient referral pathway. To prevent data leakage, splitting was performed strictly at the patient level: both eyes of any given subject were always assigned to the same partition, ensuring that no patient contributed images to more than one of the training, internal validation, and external test sets. Stratification preserved class proportions while this patient-level constraint was maintained. Each subject contributed a single imaging session; no patient provided repeated imaging over time, so temporal leakage across sessions was not possible.

### 2.4 Image preprocessing

All fundus images were resized to 512 × 512 pixels using bilinear interpolation, followed by background masking via circular field-of-view detection. To mitigate inter-device variability, a two-stage normalization was applied: Contrast-Limited Adaptive Histogram Equalization (CLAHE; clip limit 2.0, tile grid 8 × 8) followed by CycleGAN-based domain normalization. The CycleGAN was pretrained on 1,200 unpaired fundus images per device pair from the DRIVE and ODIR-5 K datasets, independent of the study cohort. Domain harmonization was validated by confirming non-significant pixel intensity distribution differences post-normalization (Kolmogorov–Smirnov test, *p* > 0.05 for all pairs). Images were normalized to zero mean and unit variance using ImageNet channel statistics.

**Figure 2:**
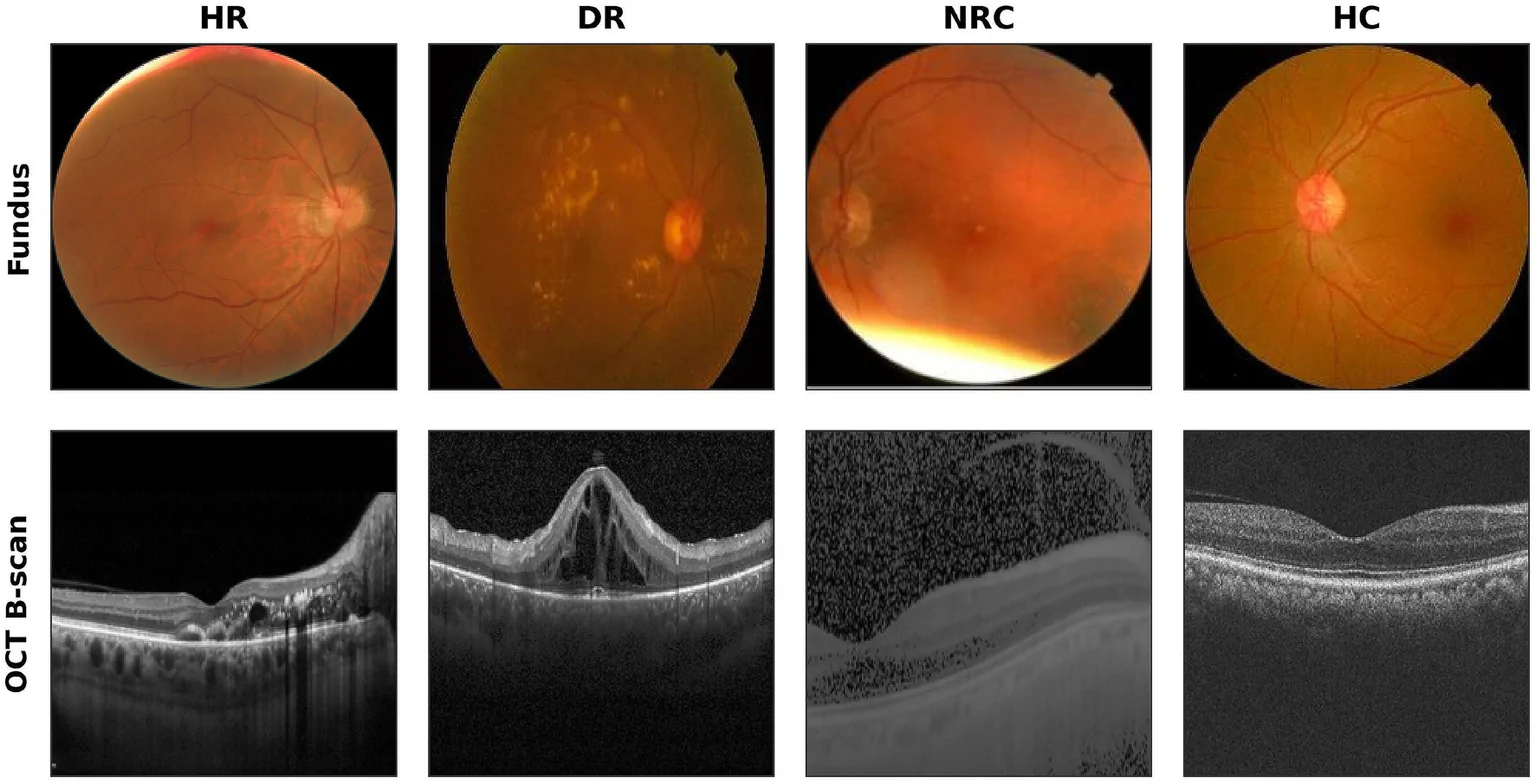
Representative preprocessed fundus photographs (top row) and corresponding OCT B-scans (bottom row) for each of the four diagnostic categories included in this study.

OCT B-scans were harmonized across devices: Topcon Triton (512 × 256 × 885 voxels), Zeiss Cirrus (512 × 128 × 1,024 voxels), and Heidelberg Spectralis (512 × 49 × 496 voxels). All volumes were resampled to a uniform isotropic resolution of 11.7 μm/pixel axially, cropped to the central 49 B-scans, and each B-scan resized to 224 × 224 pixels. Retinal layer segmentation used the Iowa Reference Algorithms, yielding nine sublayers including RNFL, GCL, IPL, INL, OPL, ONL, IS/OS, RPE, and choroid. Segmentation quality was verified on 150 randomly selected volumes (mean Dice = 0.91 ± 0.04). Figure 2 illustrates representative preprocessed fundus images and OCT B-scans for each diagnostic category. Images are shown after standardized preprocessing, including CLAHE-based contrast enhancement and CycleGAN-based domain normalization. HR = Hypertensive Retinopathy; DR = Diabetic Retinopathy; NRC = Neurodegenerative-associated Retinal Changes; HC = Healthy Controls.

### 2.5 Data augmentation and class balancing

Augmentation was applied in two sequential stages. In the first stage, online image-level augmentation was applied at each epoch: random horizontal and vertical flipping (*p* = 0.5), rotation within ±15 degrees, brightness and contrast jitter (factor range 0.8–1.2), Gaussian blur (sigma 0.1–2.0, *p* = 0.3), and random cropping with resizing to 512 × 512 (crop ratio 0.85–1.0). Mixup augmentation (*α* = 0.4) was applied at the batch level prior to the forward pass.

The second stage addressed class imbalance and was performed independently within each training fold, using only the training partition of that fold; validation and external test data were never accessed at any step. The procedure was as follows. (i) The fold’s training images were passed once through the frozen model to extract a 512-dimensional fused embedding per subject. (ii) Synthetic Minority Oversampling Technique (SMOTE) was applied in this embedding space to the minority classes (NRC and HC), interpolating between nearest neighbors to synthesize additional minority embeddings up to 85% of the majority-class count. (iii) These synthetic embeddings were not used as model inputs; instead, they were used only to derive class-balanced sampling probabilities for the subsequent training. (iv) During actual training, every forward and backward pass operated exclusively on real image data drawn according to these balanced sampling probabilities, complemented by class-weighted cross-entropy. Because steps (i)–(iv) were repeated separately for each fold on its own training data, no information from validation or test partitions could leak into the balancing procedure. After oversampling, the effective training distribution per fold was HR 657, DR 607, NRC 558, HC 558 (total 2,380) (Table 2).

**Table 2:** Class distribution before and after SMOTE-based oversampling.

Class	Original count	After balancing (per fold)
Hypertensive Retinopathy	657	657
Diabetic Retinopathy	607	607
NRC (Neurodegenerative)	360	558
Healthy Controls	263	558
Total	1,887	2,380

### 2.6 Model architecture

The proposed framework, RetinalVNG-Net (Retinal Vascular-Neuro-Glial Network), consists of three parallel encoding branches followed by a cross-modal fusion module and a multi-task output head.

The fundus branch uses RETFound (Vision Transformer Large (ViT-Large), pretrained on 1.6 million retinal images via masked autoencoding) with frozen weights for the first 18 transformer blocks, while the final 6 blocks and a lightweight adapter layer (bottleneck dimension: 64) were fine-tuned end-to-end. The OCT branch consists of two parallel sub-streams: (1) a volumetric sub-stream processing raw harmonized B-scan volumes (B × 49 × 1 × 224 × 224) via ResNet-3D-18, outputting a 256-dimensional embedding; and (2) a structural sub-stream encoding per-layer thickness maps (B × 9 × 512 × 128) via a lightweight 2D-CNN, outputting a 128-dimensional embedding. Concatenated, these form a 384-dimensional OCT representation. The clinical metadata branch encodes 11 continuous and 1 binary variable through a four-layer tabular transformer (hidden dimension: 128), producing a 128-dimensional representation.

All three branch representations are projected to a unified 512-dimensional space via linear projection layers with GELU activation. Multimodal fusion uses a cross-attention transformer block with 8 attention heads and feed-forward dimension of 2,048, followed by layer normalization and residual connection. The fused 512-dimensional representation feeds two output heads: (1) a four-class softmax classification head, and (2) an auxiliary regression head (512 → 128 → 1) generating the RBAG score.

**Figure 3:**
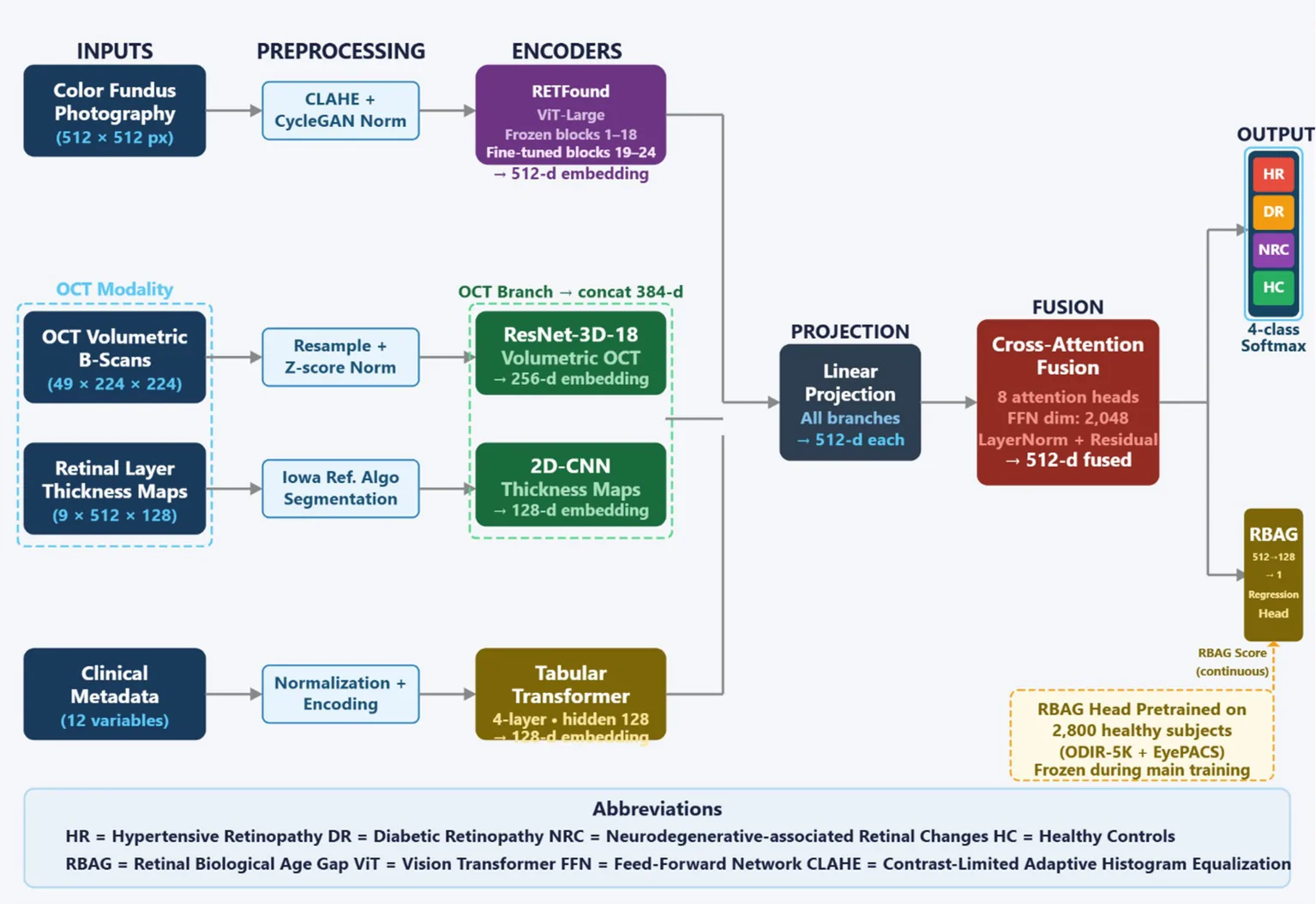
Overall workflow diagram of RetinalVNG-Net.

RBAG is defined as the signed difference between model-predicted retinal biological age and chronological age. The auxiliary regression head was pretrained on 2,800 healthy subjects from ODIR-5 K and Kaggle EyePACS datasets (independent of the study cohort), using chronological age as the regression target. Pretraining ran for 50 epochs using MSE loss with AdamW (learning rate: 5 × 10^−5^), achieving MAE of 3.2 ± 1.1 years. During primary training, the pretrained weights of the regression head were held fixed, but the auxiliary regression loss was retained and backpropagated into the shared fusion representation that feeds the head. Thus, the RBAG objective did not update the head itself; rather, it acted as an auxiliary task that regularized and shaped the shared multimodal embedding through the fusion and projection layers, which remained trainable. Figure 3 presents the overall framework workflow.

The framework integrates three parallel input streams, color fundus photographs, OCT volumetric B-scans and retinal layer thickness maps, and structured clinical metadata, through dedicated encoding branches (RETFound ViT-Large, dual-stream OCT encoder, and tabular transformer, respectively). Branch representations are projected to a unified 512-dimensional embedding space and fused via a cross-attention transformer block. The fused representation feeds two output heads: a four-class softmax classification head for disease risk stratification (HR, DR, NRC, HC) and an auxiliary regression head generating the Retinal Biological Age Gap (RBAG) score. RBAG = Retinal Biological Age Gap; HR = Hypertensive Retinopathy; DR = Diabetic Retinopathy; NRC = Neurodegenerative-associated Retinal Changes; HC = Healthy Controls.

### 2.7 Training configuration and hyperparameter optimization

The model was implemented in PyTorch 2.1 with mixed-precision training (FP16) on two NVIDIA A100 80GB GPUs with DistributedDataParallel (DDP). Total trainable parameters: approximately 105.5 million (out of 307 million total in the RETFound backbone, with 230 million frozen).

The total training loss was L_total = L_cls + *λ* · L_reg, where L_cls is class-weighted cross-entropy with label smoothing (*ε* = 0.1), L_reg is smooth L1 (Huber, *δ* = 1.0), and λ = 0.3.

Although the regression head’s own parameters were frozen, L_reg was differentiable with respect to the upstream fused representation; its gradients therefore flowed into the trainable fusion and projection layers, allowing the age-regression signal to inform the shared embedding without modifying the frozen head. This is what is meant by the regression loss contributing to training despite the head being fixed. The AdamW optimizer used a base learning rate of 1 × 10^−4^, backbone learning rate 1 × 10^−5^, and weight decay 1 × 10^−4^. A cosine annealing schedule with 10-epoch linear warmup was applied. Models were trained for a maximum of 100 epochs with early stopping (patience 15 epochs) monitoring macro-averaged AUC-ROC. Effective batch size was 64 (batch size 32, gradient accumulation 2 steps). Gradient clipping used a maximum norm of 1.0.

All hyperparameters were selected via grid search on the dedicated hyperparameter split, evaluating: learning rate ∈ {1 × 10^−3^, 1 × 10^−4^, 5 × 10^−5^}, weight decay ∈ {1 × 10^−3^, 1 × 10^−4^}, dropout ∈ {0.1, 0.3, 0.5}, and *λ* ∈ {0.1, 0.3, 0.5} (Table 3).

**Table 3:** Final hyperparameter configuration.

Hyperparameter	Value
Optimizer	AdamW
Base Learning Rate	1 × 10^−4^
Backbone Learning Rate	1 × 10^−5^
Layer-wise LR Decay Factor	0.75
Weight Decay	1 × 10^−4^
LR Schedule	Cosine Annealing with Warmup
Warmup Epochs	10
Maximum Epochs	100
Early Stopping Patience	15 epochs
Effective Batch Size	64
Gradient Clip Norm	1.0
Dropout Rate (fusion block)	0.3
Classification Loss	Weighted Cross-Entropy
Label Smoothing (ε)	0.1
Regression Loss	Smooth L1 (Huber, δ = 1.0)
Loss Weight λ	0.3
Mixed Precision	FP16
Hardware	2 × NVIDIA A100 80GB

### 2.8 Evaluation metrics

Model performance was evaluated using: AUC-ROC (one-vs-rest macro averaging), accuracy, sensitivity, specificity, precision, and F1-score, computed per class and as macro-averaged values. Confusion matrices were generated for both evaluation sets. t-SNE was applied to 512-dimensional fused embeddings (perplexity = 30, 1,000 iterations, seed = 42). For explainability, Attention Rollout was used for the ViT-based fundus branch, and Grad-CAM was applied at the final convolutional layer of the 3D-CNN OCT branch. Calibration was assessed using Expected Calibration Error (ECE) and Brier Score; Hosmer–Lemeshow goodness-of-fit test was used per class.

### 2.9 Baseline and comparative architecture evaluation

RetinalVNG-Net was compared against twelve baseline and ablation models (Table 4): ResNet-50, EfficientNet-B4, and VGG-16 (fundus, CNN); RETFound fine-tuned for four-class classification (fundus, ViT); ResNet-3D-18 (OCT B-scans only); ResNet-3D-18 + 2D-CNN (dual-stream OCT); Random Forest (clinical metadata); concatenation fusion (fundus + full OCT); addition fusion (fundus + full OCT); RetinalVNG-Net without metadata; RetinalVNG-Net without RBAG; and RetinalVNG-Net with concatenation fusion instead of cross-attention. All models were trained under identical data splits, augmentation, balancing, and evaluation protocols. Hyperparameters for all deep learning baselines were optimized on the same dedicated split.

**Table 4:** Summary of all compared architectures.

Model	Modality	Architecture	Fusion strategy
ResNet-50	Fundus	CNN	—
EfficientNet-B4	Fundus	CNN	—
VGG-16	Fundus	CNN	—
RETFound (fine-tuned)	Fundus	ViT-Large	—
ResNet-3D-18	OCT (B-scans only)	3D-CNN	—
ResNet-3D-18 + 2D-CNN	OCT (full)	Dual-stream CNN	Concatenation
Random Forest	Clinical Metadata	Ensemble ML	—
Concat Fusion	Fundus + OCT (full)	RETFound + Full OCT	Concatenation
Addition Fusion	Fundus + OCT (full)	RETFound + Full OCT	Element-wise Addition
VNG-Net (no metadata)	Fundus + OCT	Full OCT + RETFound	Cross-Attention
VNG-Net (no RBAG)	Fundus + OCT + Metadata	Full Architecture	Cross-Attention
VNG-Net (concat fusion)	Fundus + OCT + Metadata	Full Architecture	Concatenation
RetinalVNG-Net (proposed)	Fundus + OCT + Metadata	Full Architecture	Cross-Attention + RBAG

### 2.10 Statistical analysis

All statistical analyses were performed using Python 3.10 (scipy 1.11, statsmodels 0.14) and R 4.3. Continuous variables are reported as mean ± SD or median with IQR, depending on normality assessed by the Shapiro–Wilk test. AUC values are reported with 95% CIs from 2,000 bootstrap iterations. Pairwise AUC comparisons used DeLong’s method with Bonferroni correction. McNemar’s test compared classification accuracy between models. Subgroup analyses were stratified by age group, sex, and imaging device. Interaction terms were tested using logistic regression.

RBAG was analyzed using Pearson and Spearman correlation against disease-specific severity indices. Multivariable logistic regression assessed independent RBAG contribution after adjusting for age, sex, systolic blood pressure, HbA1c, and serum creatinine. Calibration was assessed using Hosmer–Lemeshow test, ECE, and Brier Score.

### 2.11 Implementation environment and computational resources

All experiments were implemented in Python 3.10 using PyTorch 2.1 (CUDA 12.1). Key libraries: timm (v0.9.12) for RETFound; torchio 0.19 for OCT volumes; OpenCV 4.8 and scikit-image 0.22 for preprocessing; Albumentations 1.3 for augmentation; vit-explain (v0.1.0) for Attention Rollout; pytorch-grad-cam (v1.4.8) for Grad-CAM; scikit-learn 1.3 for t-SNE; pROC (R 4.3) for DeLong’s test. Hardware: two NVIDIA A100 80GB GPUs, Intel Xeon Gold 6338 CPU (32 cores), 512 GB RAM, Ubuntu 22.04 LTS. Average training time per fold: approximately 6.4 h; total experimental runtime: approximately 140 GPU-hours. At inference, a complete multimodal forward pass (fundus, dual-stream OCT, and metadata) required approximately 0.9 s per subject on a single A100 GPU, with the volumetric OCT sub-stream accounting for the largest share of this time. On a CPU-only configuration the same pass required approximately 10 s, the increase being driven by the 3D convolutional OCT branch. Peak GPU memory at inference was approximately 9 GB. Prediction latency is therefore not a barrier to clinical use where GPU acceleration is available.

## 3 Results

### 3.1 Participant characteristics

A total of 3,481 subjects were initially screened. Following pre-screening, 549 were excluded (201 for age outside range, 183 for missing imaging, 165 for incomplete metadata). Of the remaining 2,932, an additional 192 were excluded due to co-occurrence of more than one target condition (Center A = 87, Center B = 64, Center C = 41), yielding a final analyzable cohort of 2,740 subjects. The development pool comprised 2,220 subjects from Centers A and B, of whom 333 were reserved for hyperparameter optimization and 1,887 formed the cross-validation pool. Center C contributed 520 subjects as the external test set. Demographic characteristics were well-matched across centers: mean age ranged from 56.9 to 58.3 years and female proportion from 49.8 to 52.1%, with no statistically significant differences (one-way ANOVA, *p* = 0.61 for age; chi-square, *p* = 0.74 for sex). Group-wise baseline characteristics across the four diagnostic categories are reported in Table 5. As expected, the NRC group was significantly older than the other groups (mean 64.7 years; *p* < 0.001), and the HR and DR groups showed the expected elevations in blood pressure and glycemic indices, respectively. Because age can influence OCT-derived thickness, age was retained as a covariate in the RBAG regression analyses, and subgroup analysis stratified by age (Section 3.10) confirmed that model performance was stable across age strata, reducing the likelihood that group age differences alone accounted for discrimination. Complete cohort characteristics are presented in Table 1.

**Table 5:** Group-wise baseline characteristics across the four diagnostic categories (pooled across all centers).

Variable	HR (*n* = 905)	DR (*n* = 835)	NRC (*n* = 495)	HC (*n* = 505)	*p*-value
Age, mean ± SD (years)	59.1 ± 10.8	58.4 ± 11.2	64.7 ± 9.6	54.2 ± 11.9	<0.001
Sex, % female	50.8	48.9	52.3	51.7	0.62
Systolic BP, mmHg	152 ± 16	134 ± 14	129 ± 13	124 ± 11	<0.001
Diastolic BP, mmHg	94 ± 10	82 ± 9	79 ± 8	77 ± 8	<0.001
Fasting glucose, mmol/L	5.4 ± 0.9	8.9 ± 2.1	5.3 ± 0.8	5.1 ± 0.7	<0.001
HbA1c, %	5.7 ± 0.5	8.4 ± 1.3	5.6 ± 0.5	5.4 ± 0.4	<0.001
Serum creatinine, μmol/L	84 ± 18	89 ± 21	80 ± 16	76 ± 14	<0.001

Continuous variables compared by one-way ANOVA or Kruskal–Wallis test; sex compared by chi-square test. HR, Hypertensive Retinopathy; DR, Diabetic Retinopathy; NRC, Neurodegenerative-associated Retinal Changes; HC, Healthy Controls.

### 3.2 Model performance: per-class metrics across all evaluation sets

Per-class and macro-averaged performance of RetinalVNG-Net across training folds, internal validation folds, and external test set is reported in Table 6. On the training partition, macro-averaged AUC-ROC reached 0.984 ± 0.004. Internal validation performance was 0.957 (95% CI: 0.941–0.973; SD ± 0.008), indicating a controlled generalization gap (training-to-validation AUC difference: 0.027, 95% CI: 0.018–0.036) without evidence of severe overfitting. External test performance of 0.944 (95% CI: 0.922–0.958) confirmed generalizability (validation-to-external AUC difference: 0.013, 95% CI: 0.004–0.022). Inter-fold variability was low (SD ≤ 0.021). The highest per-class AUC was achieved for hypertensive retinopathy across all evaluation sets (external AUC: 0.963, 95% CI: 0.941–0.979); the lowest for neurodegenerative-associated retinal changes (external AUC: 0.924, 95% CI: 0.897–0.947), reflecting greater morphological similarity between early neuroretinal thinning and normal aging.

**Table 6:** Per-class and macro-averaged performance of RetinalVNG-Net.

Split	Class	AUC-ROC	Sensitivity	Specificity/Precision/F1
Training (mean ± SD)	HR	0.991 ± 0.003	0.974 ± 0.006	Spec 0.983/Prec 0.969/F1 0.971
	DR	0.988 ± 0.004	0.968 ± 0.007	Spec 0.979/Prec 0.963/F1 0.965
	NRC	0.976 ± 0.006	0.951 ± 0.009	Spec 0.968/Prec 0.946/F1 0.948
	HC	0.981 ± 0.005	0.959 ± 0.008	Spec 0.974/Prec 0.954/F1 0.956
	Macro	0.984 ± 0.004	0.963 ± 0.007	Spec 0.976/Prec 0.958/F1 0.960
Validation (mean ± SD)	HR	0.968 ± 0.007	0.931 ± 0.012	Spec 0.954/Prec 0.921/F1 0.926
	DR	0.961 ± 0.009	0.921 ± 0.014	Spec 0.948/Prec 0.914/F1 0.917
	NRC	0.931 ± 0.014	0.884 ± 0.019	Spec 0.929/Prec 0.879/F1 0.881
	HC	0.944 ± 0.011	0.906 ± 0.016	Spec 0.941/Prec 0.901/F1 0.903
	Macro	0.957 ± 0.008	0.913 ± 0.011	Spec 0.941/Prec 0.906/F1 0.908
External test	HR	0.963 (0.941–0.979)	0.926	Spec 0.949/Prec 0.918/F1 0.922
	DR	0.951 (0.928–0.968)	0.912	Spec 0.941/Prec 0.907/F1 0.909
	NRC	0.924 (0.897–0.947)	0.871	Spec 0.921/Prec 0.863/F1 0.867
	HC	0.938 (0.912–0.958)	0.894	Spec 0.934/Prec 0.889/F1 0.891
	Macro	0.944 (0.922–0.958)	0.901	Spec 0.936/Prec 0.894/F1 0.897

HR, Hypertensive Retinopathy; DR, Diabetic Retinopathy; NRC, Neurodegenerative-associated Retinal Changes; HC, Healthy Controls. External test AUC reported with 95% bootstrap Confidence Interval (CI) (2,000 iterations).

Calibration metrics are reported in Table 7. All Hosmer–Lemeshow *p*-values exceeded 0.05, and ECE remained below 0.044 across all classes and evaluation sets, confirming satisfactory probability calibration. These figures should be interpreted in light of two factors that could inflate apparent performance: the inclusion of clinical metadata closely tied to the target diseases, and the partial dependence of the NRC label on OCT thickness criteria that overlap with model inputs. Both were examined directly. Removing clinical metadata reduced external macro-AUC only modestly to 0.929 (Table 8; per-class detail in Supplementary Table S1), and removing the OCT thickness features used in the NRC definition reduced external NRC AUC from 0.924 to 0.902, with a diagnosis-only NRC definition yielding 0.883 (Section 3.11). Performance therefore persisted under both stricter conditions, though the headline figures should be read as upper-bound estimates obtained under a single-label, comorbidity-excluded design.

**Table 7:** Calibration metrics (ECE, Brier Score, Hosmer–Lemeshow *p*) per class and evaluation set.

Split	Class	ECE	Brier score	H–L *p*-value
Training (mean ± SD)	HR	0.018 ± 0.004	0.041 ± 0.006	>0.05
	DR	0.020 ± 0.004	0.045 ± 0.007	>0.05
	NRC	0.023 ± 0.005	0.051 ± 0.008	>0.05
	HC	0.019 ± 0.004	0.043 ± 0.006	>0.05
Validation (mean ± SD)	HR	0.029 ± 0.006	0.068 ± 0.009	>0.05
	DR	0.031 ± 0.005	0.073 ± 0.008	>0.05
	NRC	0.034 ± 0.007	0.079 ± 0.011	>0.05
	HC	0.030 ± 0.006	0.071 ± 0.009	>0.05
External test	HR	0.034	0.076	0.31
	DR	0.037	0.082	0.27
	NRC	0.043	0.091	0.19
	HC	0.036	0.079	0.24

**Table 8:** Macro-averaged performance of all thirteen architectures across all evaluation sets.

Model	Split	AUC (95% CI)	Sensitivity	Specificity	F1	*p*-value
ResNet-50 (Fundus)	Training	0.934 ± 0.011	0.901	0.929	0.898	<0.001
	Validation	0.871 ± 0.014	0.836	0.874	0.832	
	External	0.858 (0.831–0.882)	0.822	0.871	0.831	
EfficientNet-B4 (Fundus)	Training	0.941 ± 0.010	0.909	0.936	0.906	<0.001
	Validation	0.883 ± 0.012	0.849	0.884	0.846	
	External	0.871 (0.845–0.894)	0.836	0.882	0.847	
VGG-16 (Fundus)	Training	0.928 ± 0.013	0.893	0.921	0.890	<0.001
	Validation	0.854 ± 0.016	0.818	0.861	0.815	
	External	0.843 (0.815–0.868)	0.807	0.858	0.819	
RETFound (Fundus)	Training	0.961 ± 0.008	0.929	0.951	0.926	<0.001
	Validation	0.914 ± 0.010	0.881	0.906	0.877	
	External	0.901 (0.876–0.924)	0.869	0.904	0.878	
ResNet-3D-18 (OCT)	Training	0.947 ± 0.010	0.914	0.939	0.911	<0.001
	Validation	0.889 ± 0.013	0.856	0.887	0.852	
	External	0.877 (0.851–0.900)	0.843	0.886	0.853	
ResNet-3D-18 + 2D-CNN (OCT)	Training	0.958 ± 0.008	0.924	0.948	0.921	<0.001
	Validation	0.908 ± 0.011	0.871	0.901	0.867	
	External	0.896 (0.871–0.919)	0.861	0.899	0.872	
Random Forest (Metadata)	Training	0.891 ± 0.014	0.857	0.884	0.854	<0.001
	Validation	0.824 ± 0.018	0.789	0.831	0.785	
	External	0.812 (0.783–0.839)	0.774	0.831	0.784	
Concat Fusion (Fundus+OCT)	Training	0.971 ± 0.006	0.938	0.958	0.935	0.003
	Validation	0.934 ± 0.009	0.899	0.924	0.896	
	External	0.922 (0.899–0.941)	0.887	0.921	0.899	
Addition Fusion (Fundus+OCT)	Training	0.969 ± 0.007	0.936	0.956	0.933	0.001
	Validation	0.931 ± 0.010	0.896	0.921	0.892	
	External	0.918 (0.894–0.938)	0.882	0.917	0.894	
VNG-Net (no metadata)	Training	0.975 ± 0.005	0.944	0.963	0.941	0.018
	Validation	0.941 ± 0.008	0.906	0.931	0.903	
	External	0.929 (0.907–0.948)	0.893	0.928	0.906	
VNG-Net (no RBAG)	Training	0.979 ± 0.005	0.949	0.967	0.946	0.042
	Validation	0.948 ± 0.008	0.913	0.936	0.910	
	External	0.936 (0.914–0.954)	0.896	0.931	0.889	
VNG-Net (concat fusion)	Training	0.973 ± 0.006	0.941	0.961	0.938	0.009
	Validation	0.938 ± 0.009	0.903	0.927	0.900	
	External	0.926 (0.903–0.945)	0.889	0.924	0.902	
RetinalVNG-Net (proposed)	Training	0.984 ± 0.004	0.963	0.976	0.960	—
	Validation	0.957 ± 0.008	0.913	0.941	0.908	
	External	0.944 (0.922–0.958)	0.901	0.936	0.897	

Values are macro-averaged. Training and validation reported as mean ± SD over five folds; external test AUC with 95% bootstrap CI (2,000 iterations). *p*-values from DeLong’s test (external test AUC vs. RetinalVNG-Net) with Bonferroni correction.

### 3.3 ROC curves

**Figure 4:**
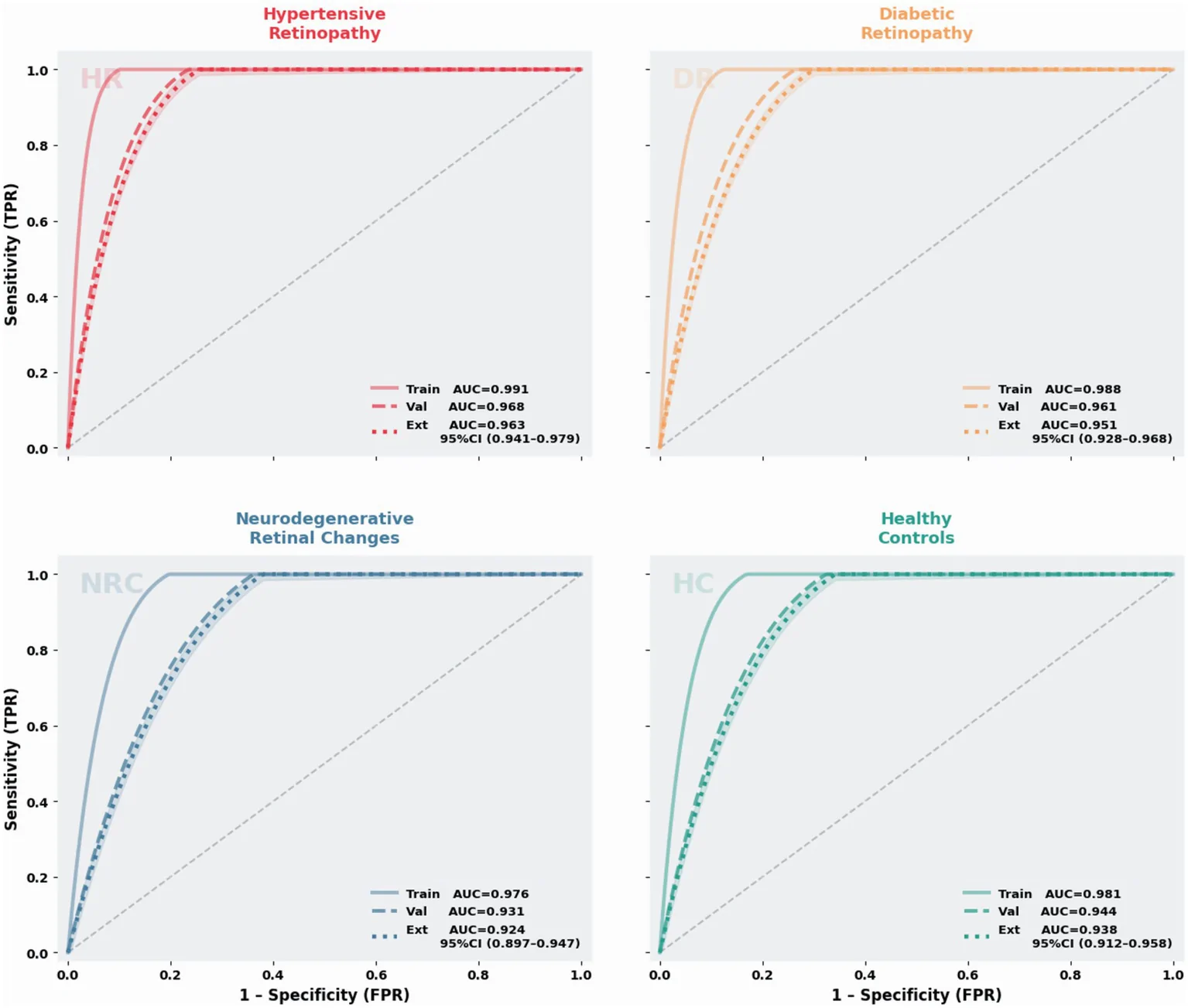
ROC curves for each of the four diagnostic classes (HR, DR, NRC, HC) displayed across three evaluation sets: training folds (solid line), internal validation folds (dashed line, mean over five folds), and external test set (dotted line with shaded 95% confidence interval estimated via 2,000 bootstrap iterations).

ROC curves for all four diagnostic classes across all three evaluation sets are presented in Figure 4. External test AUC ranged from 0.924 (NRC) to 0.963 (HR), with narrow bootstrap CIs confirming stability. The visual proximity of training, validation, and external test curves across all classes confirms the absence of severe overfitting and supports cross-device generalizability.

### 3.4 Confusion matrix

**Figure 5:**
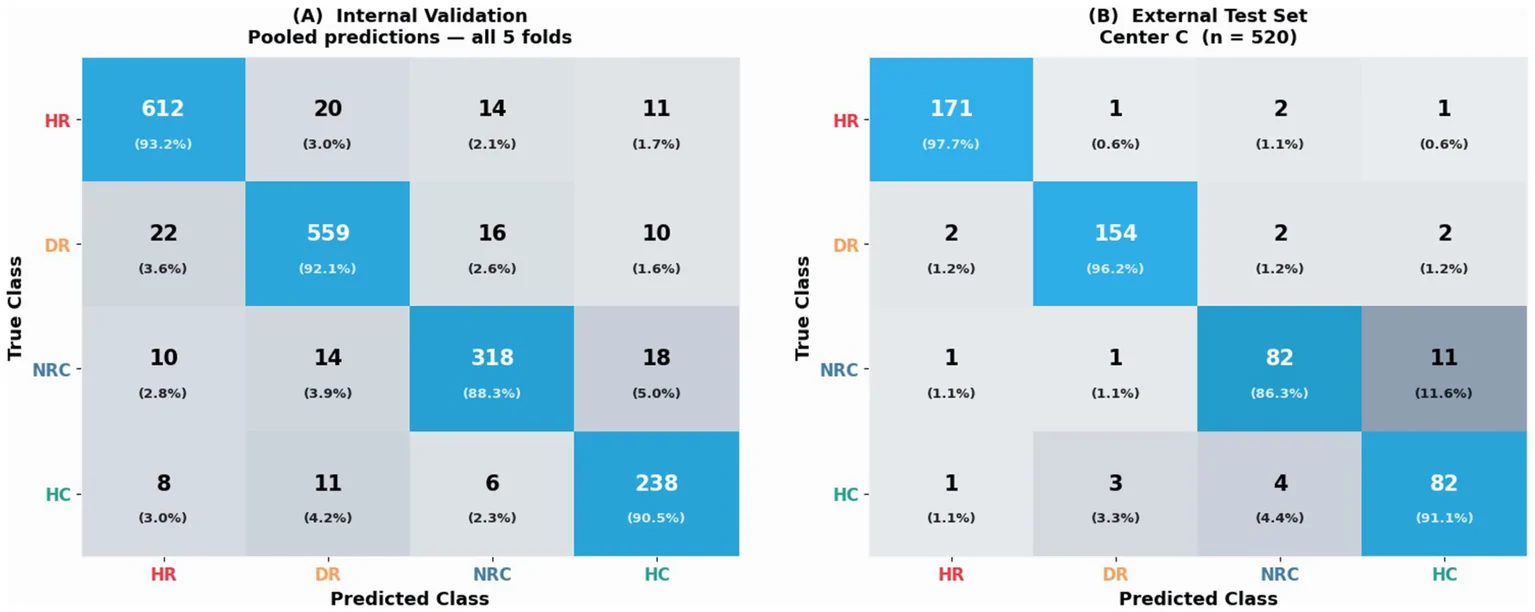
Confusion matrices of RetinalVNG-Net on **(A)** the internal validation set (pooled predictions aggregated across all five validation folds) and **(B)** the external test set (Center C, *n* = 520). Each cell displays both the absolute subject count and the row-normalized percentage (count/row total × 100%) to account for class size differences. Diagonal cells indicate correct classifications. Off-diagonal cells indicate misclassifications. Color intensity scales with the normalized percentage within each row. HR, Hypertensive Retinopathy; DR, Diabetic Retinopathy; NRC, Neurodegenerative-associated Retinal Changes; HC, Healthy Controls.

Confusion matrices for both the internal validation set and external test set are presented in Figure 5. On the external test set, the most frequent misclassification was between NRC and healthy controls (11 of 95 NRC subjects, 11.6%), consistent with morphological overlap between early neuroretinal thinning and normal aging. HR demonstrated the fewest errors (4 of 175 subjects, 2.3%). HR–DR cross-confusion was minimal (3 subjects, 1.7%), indicating effective discrimination between two conditions sharing a microvascular substrate.

### 3.5 t-SNE embedding visualization

**Figure 6:**
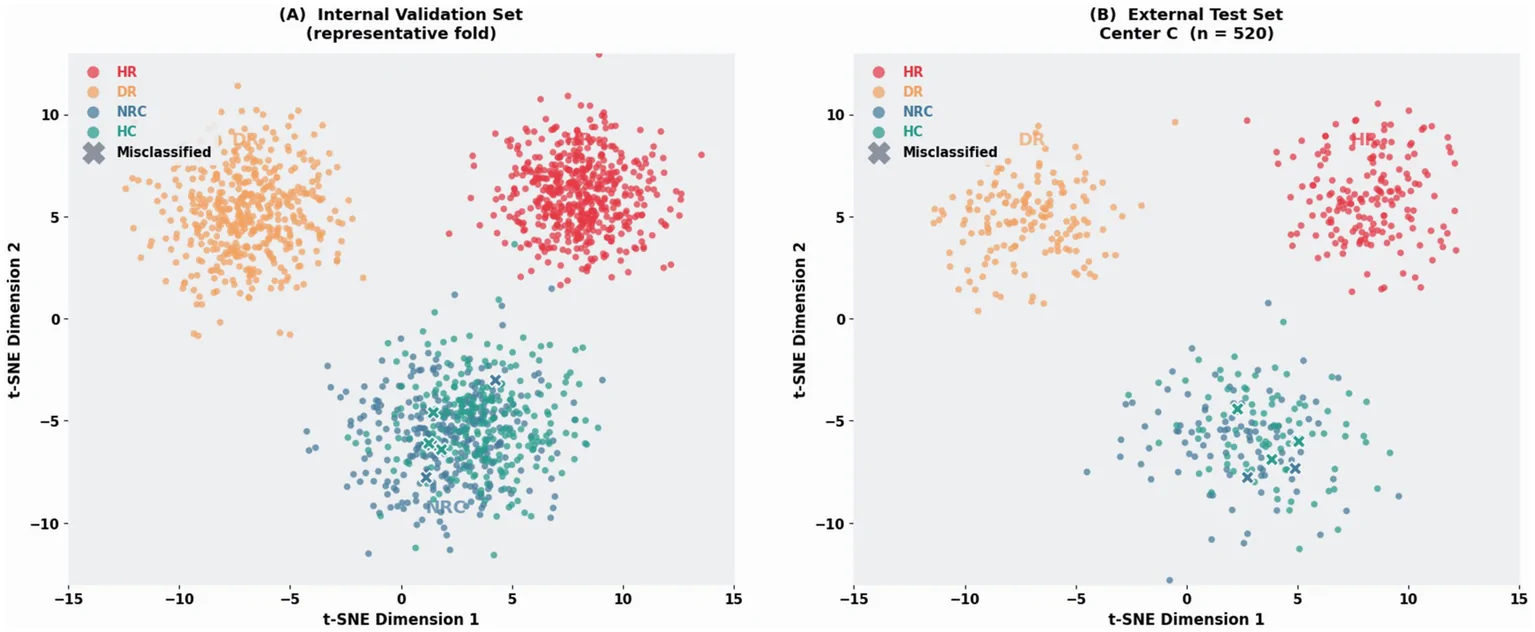
t-SNE visualization of 512-dimensional fused embeddings from RetinalVNG-Net on **(A)** the internal validation set (mean fold shown, representative of all five folds) and **(B)** the external test set (Center C, *n* = 520). Embeddings computed using perplexity = 30, 1,000 iterations, and random seed = 42 for reproducibility. This projection is exploratory and intended for qualitative illustration only; cluster appearance may vary with t-SNE parameter settings.

As an exploratory visualization, t-SNE applied to the 512-dimensional fused embeddings (Figure 6) suggested apparent clustering of HR and DR in both evaluation sets, with partial overlap between NRC and healthy controls, broadly concordant with the confusion matrix. Because t-SNE is qualitative and sensitive to perplexity and initialization, these projections are presented for illustration only and are not interpreted as quantitative evidence of class separability. Misclassified subjects predominantly originate at the NRC–HC boundary, reflecting the biological continuum between preclinical neurodegeneration and normal retinal aging.

### 3.6 Explainability: attention rollout and grad-CAM

**Figure 7:**
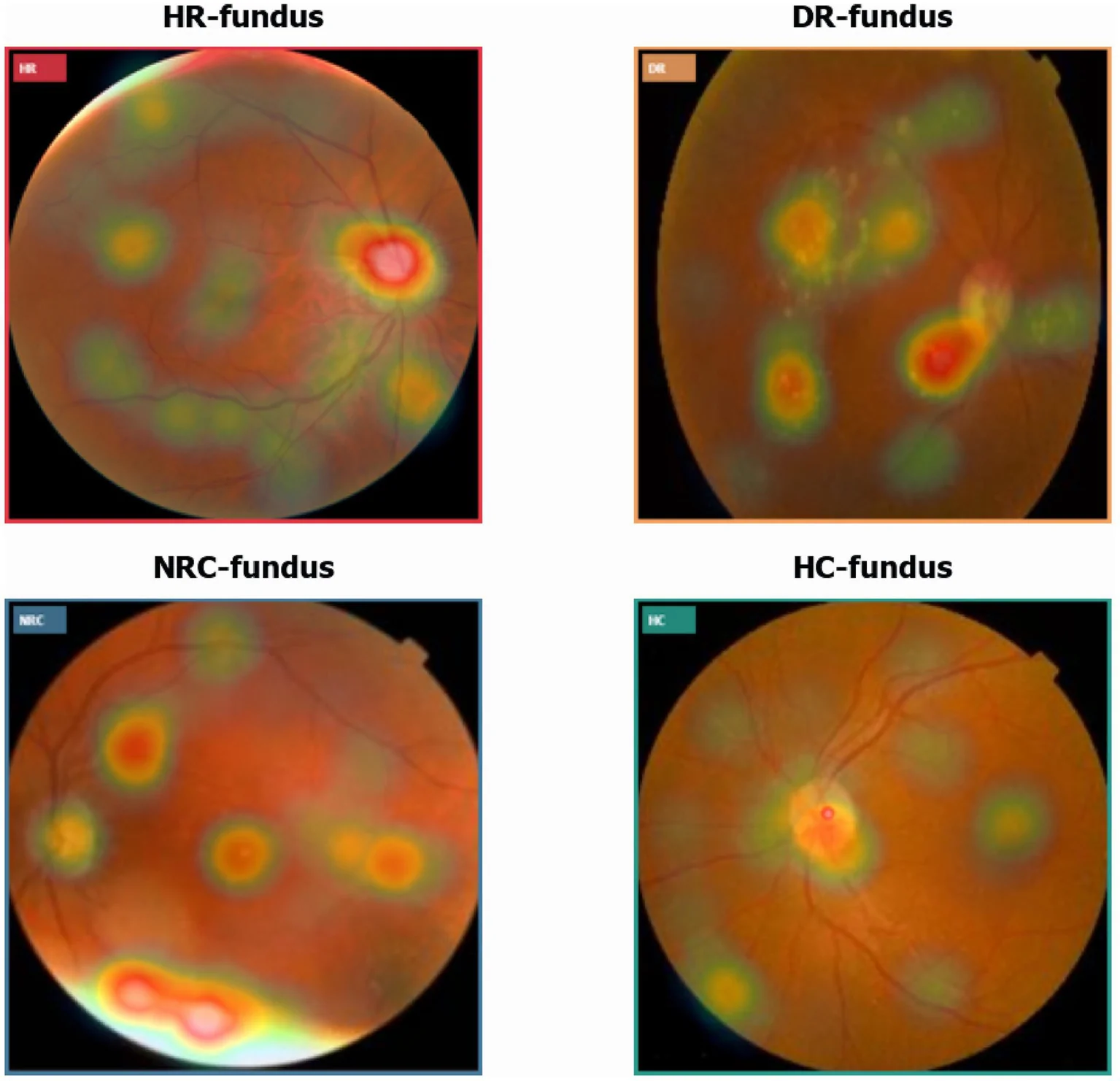
Attention rollout maps from the RETFound ViT-Large fundus branch overlaid on original preprocessed fundus images for each diagnostic class on the external test set.

**Figure 8:**
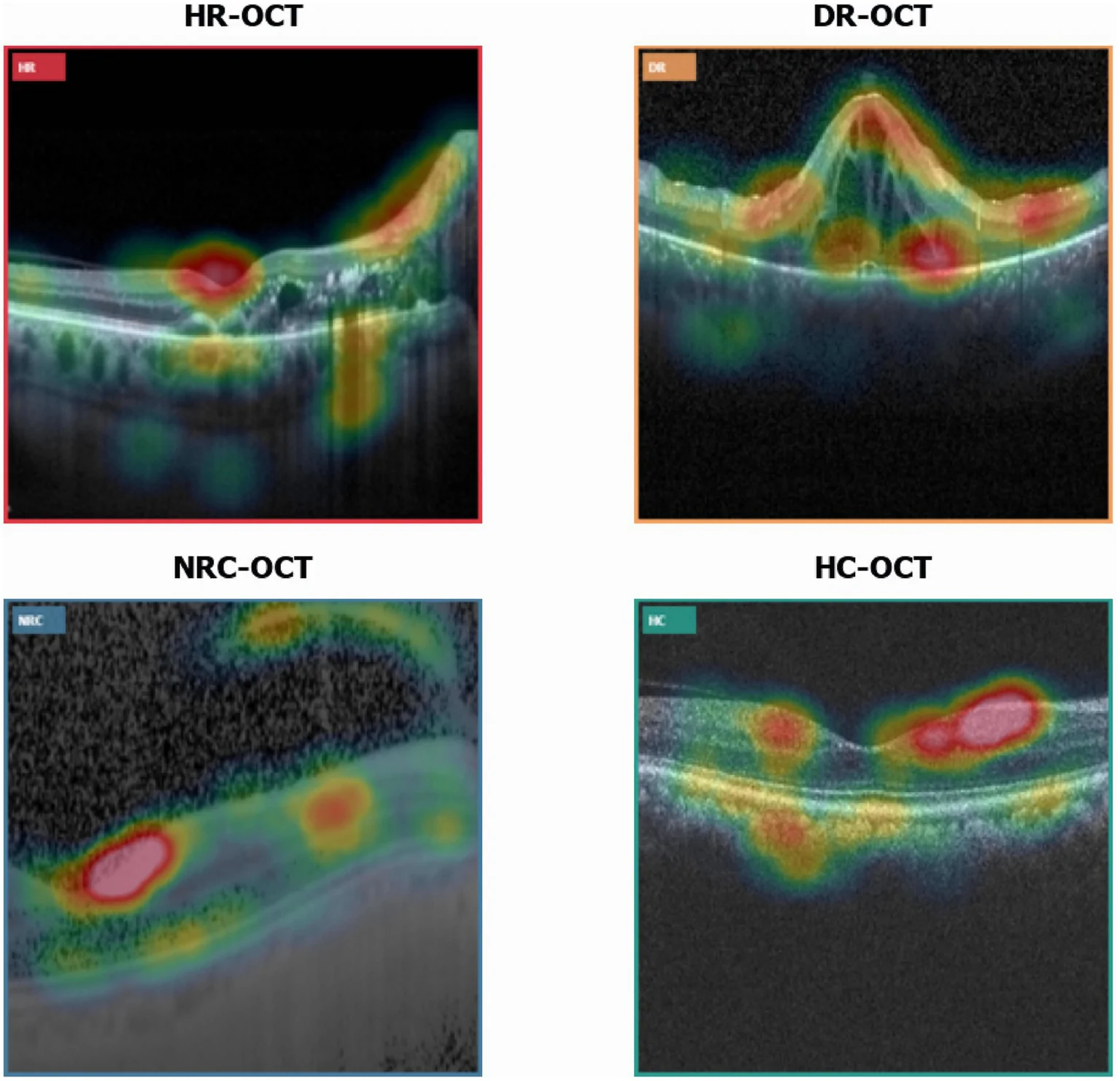
Grad-CAM activation maps from the 3D-CNN OCT branch (ResNet-3D-18) for each diagnostic class on the external test set.

Attention Rollout maps (Figure 7) and Grad-CAM activation maps (Figure 8) are presented for representative external test cases across all four classes. For HR, attention localized to arteriolar segments, arteriovenous crossing points, and the optic disc margin. For DR, high-attention regions corresponded to the posterior pole and mid-peripheral arcades. For NRC, attention concentrated on the peripapillary region and macular ganglion cell complex. Misclassified NRC cases showed diffuse attention without peripapillary concentration. Grad-CAM maps were computed on the B-scan yielding maximum class activation per volume. Qualitative review by two senior retinal specialists confirmed clinical plausibility of all class-specific activation patterns in correctly classified cases.

### 3.7 Comparative performance against baselines

RetinalVNG-Net significantly outperformed all twelve comparative architectures on the external test set. RETFound fine-tuned on fundus alone achieved AUC 0.901 (95% CI: 0.876–0.924; ΔAUC = +0.043, 95% CI: 0.029–0.057; DeLong’s Z = 4.82, *p* < 0.001). Concatenation fusion achieved AUC 0.922 (95% CI: 0.899–0.941; ΔAUC = +0.022, 95% CI: 0.011–0.033; DeLong’s Z = 3.21, *p* = 0.003) and addition fusion 0.918 (95% CI: 0.894–0.938; ΔAUC = +0.026, 95% CI: 0.015–0.037; DeLong’s Z = 3.47, *p* = 0.001). Random Forest on metadata alone achieved AUC 0.812. McNemar’s test confirmed significant accuracy differences against all twelve baselines (all *p* < 0.05 after Bonferroni correction). Complete results are in Table 8.

### 3.8 Ablation study

Systematic ablation confirmed the independent and additive contribution of each architectural component. A per-class breakdown of metadata-only versus imaging-only performance is provided in Supplementary Table S1. The metadata-only Random Forest reached an external macro-AUC of 0.812, with its highest values for HR and DR, the two conditions most directly tied to blood pressure and glycemic variables, and lower values for NRC and HC. Across all classes the imaging-only configuration substantially exceeded metadata-only performance, indicating that retinal imaging rather than systemic clinical variables carried the dominant discriminative signal, with metadata refining rather than driving the predictions. Replacement of cross-attention with concatenation reduced AUC by 0.018 (95% CI: 0.005–0.031; DeLong’s Z = 2.71, *p* = 0.009). Removal of the RBAG head reduced AUC by 0.008 (95% CI: 0.001–0.016, *p* = 0.042) and macro F1 by 0.008, reflecting the regularizing benefit of the multi-task training objective. Addition of the OCT thickness map sub-stream over raw B-scans alone yielded ΔAUC = +0.019 (95% CI: 0.009–0.029, *p* < 0.001).

### 3.9 RBAG score analysis

RBAG demonstrated significant positive correlation with disease severity on the external test set: Pearson *r* = 0.71 (95% CI: 0.62–0.78, *p* < 0.001) with Keith–Wagener–Barker grade for HR; *r* = 0.68 (95% CI: 0.59–0.76, *p* < 0.001) with ETDRS severity for DR; *r* = −0.63 (95% CI: −0.71 to −0.53, *p* < 0.001) with MoCA cognitive score for NRC. Spearman correlations were consistent (*ρ* = 0.69, 0.66, −0.61). These patterns were replicated in the internal validation set (*r* = 0.69, 0.65, −0.61; all *p* < 0.001).

**Figure 9:**
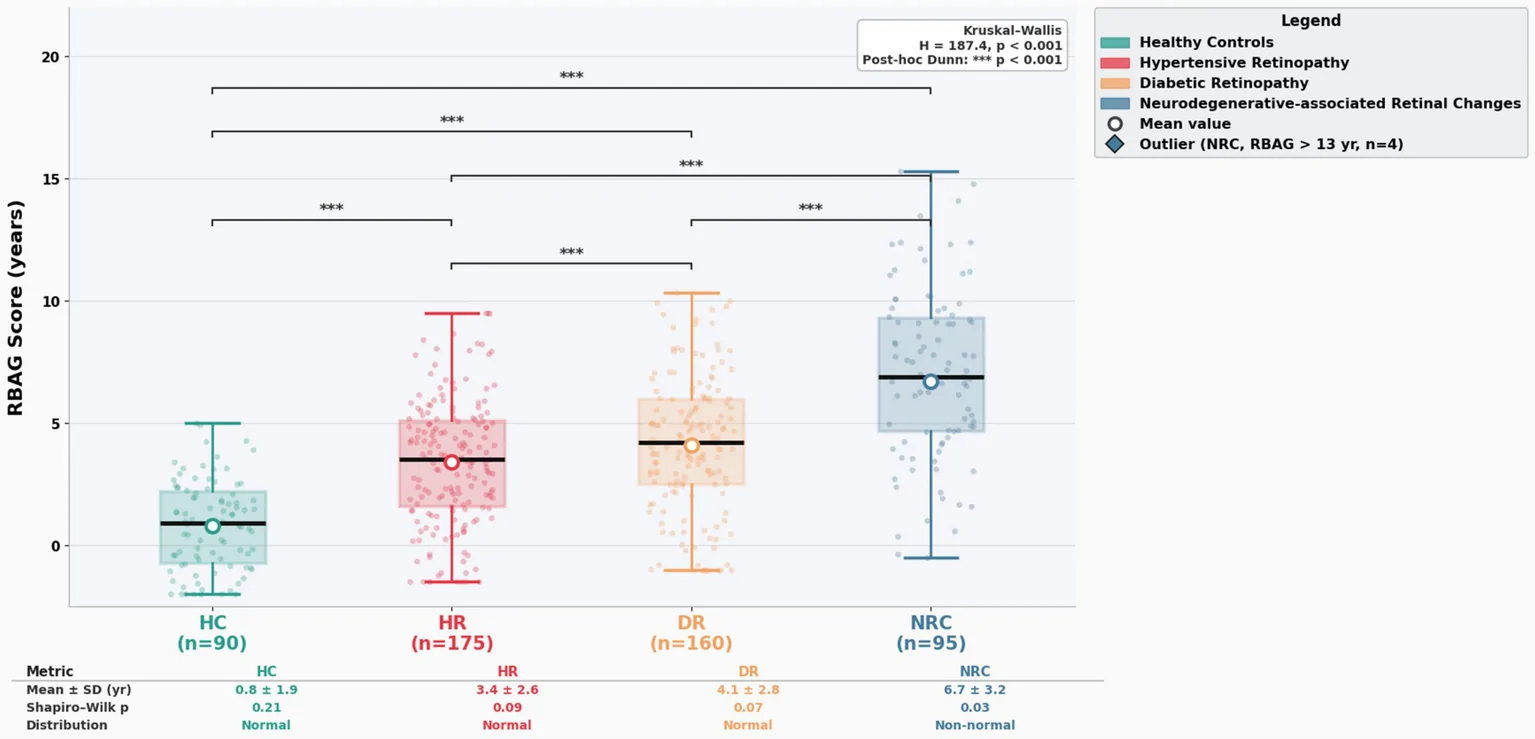
Distribution of Retinal Biological Age Gap (RBAG) scores by diagnostic category on the external test set (Center C, *n* = 520).

In multinomial logistic regression adjusting for age, sex, systolic blood pressure, HbA1c, and serum creatinine, RBAG remained an independent predictor across all three disease categories: HR (OR per unit increase: 1.28, 95% CI: 1.14–1.44, *p* < 0.001), DR (OR: 1.31, 95% CI: 1.17–1.47, *p* < 0.001), and NRC (OR: 1.41, 95% CI: 1.26–1.58, *p* < 0.001). Mean RBAG showed a progressive gradient: healthy controls +0.8 ± 1.9 years; HR + 3.4 ± 2.6 years; DR + 4.1 ± 2.8 years; NRC + 6.7 ± 3.2 years. RBAG distribution is shown in Figure 9.

Box plots display median, interquartile range, and 1.5 × IQR whiskers; individual data points are overlaid as jittered dots. Group differences were assessed using the Kruskal–Wallis test (H = 187.4, *p* < 0.001) with post-hoc Dunn correction for pairwise comparisons. All pairwise comparisons were statistically significant (*** *p* < 0.001). Outliers in the NRC group (RBAG > + 13 years, *n* = 4) represent subjects with advanced-stage neurodegeneration confirmed by both clinical diagnosis and severe pRNFL thinning (< 2nd percentile in ≥ 4 sectors), and are retained in all analyses.

### 3.10 Subgroup and device-stratified analysis

Model performance was consistent across all pre-specified subgroups. External AUC did not differ significantly by sex (males: 0.941, females: 0.947; *p* = 0.43) or by age stratum (< 50 years: 0.938, 50–65 years: 0.946, > 65 years: 0.941; *p* = 0.61). No significant interaction between subgroup variables and diagnostic class prediction was identified (all interaction *p* > 0.10). Per-center internal validation AUC was 0.959 ± 0.009 for Center A and 0.954 ± 0.010 for Center B (*p* = 0.38), confirming effective CycleGAN-based domain harmonization. External test performance of 0.944 on Center C (Canon CR-2; Heidelberg Spectralis OCT) represents device-specific performance on an unseen imaging platform.

### 3.11 NRC label sensitivity analysis

To address the potential circularity between the OCT-based component of the NRC definition and the OCT model inputs, we performed two sensitivity analyses on the external test set. First, we removed the retinal layer thickness map sub-stream, the input most directly aligned with the pRNFL and mGCIPL criteria used to define NRC. Under this configuration, external NRC AUC decreased from 0.924 to 0.902 (ΔAUC = −0.022, 95% CI: 0.008–0.036), indicating that the model retained substantial discriminative ability without the thickness features embedded in the label definition. Second, we relabeled the NRC group using neurological diagnosis alone, that is, confirmed Alzheimer’s or Parkinson’s disease, discarding the OCT thinning criterion entirely, and retrained the model under identical splits and protocols. Under this diagnosis-only definition, external NRC AUC was 0.883 (95% CI: 0.851–0.911). The modest reduction relative to the original definition shows that retinal imaging predicted the clinical neurodegenerative diagnosis independently of the OCT thresholds used in the original label, supporting the conclusion that performance was not an artifact of label leakage.

## 4 Discussion

The fundus branch of RetinalVNG-Net builds on RETFound, a vision transformer pretrained via masked autoencoding on 1.6 million unlabelled retinal images, which established the foundation model paradigm for retinal AI and demonstrated generalizable representations across both ocular and systemic disease tasks including incident heart failure and myocardial infarction prediction. The present study extends this framework in a distinct direction: rather than deploying RETFound as a standalone classifier, we embed it as one branch within a multimodal cross-attention architecture, pairing it with a dual-stream OCT encoder and a tabular transformer for clinical metadata. The resulting ΔAUC gain of +0.043 over RETFound fine-tuned on fundus alone (AUC 0.901 vs. 0.944, *p* < 0.001) demonstrates that the foundation model backbone, while powerful in isolation, realizes substantially greater diagnostic potential when its representations are integrated with complementary structural and clinical information through learned cross-modal attention.

### 4.1 The RBAG biomarker in the context of retinal biological age research

The Retinal Biological Age Gap introduced in this study participates in a rapidly maturing literature. The foundational concept, that deep learning-derived retinal age estimates encode clinically meaningful biological information beyond chronological age, was established by Hu et al. (23), who showed in UK Biobank data that an elevated retinal age gap was significantly associated with future Parkinson’s disease incidence. Nusinovici et al. (24), subsequently developed RetiPhenoAge, demonstrating robust prediction of morbidity and mortality outcomes across UK Biobank, SEED, and AREDS cohorts spanning multiple ethnic populations. Most recently, Sim et al. (5) reported that retinal aging biomarkers predicted longitudinal cognitive decline and incident dementia with UK Biobank replication confirming 12-year incident dementia prediction, the longest prospective validation of a retinal biological age marker for neurodegeneration to date.

The RBAG scores in our study demonstrate correlations consistent in magnitude with these epidemiological findings: *r* = 0.71 with Keith–Wagener–Barker grade for HR, *r* = 0.68 with ETDRS severity for DR, and *r* = −0.63 with MoCA cognitive score for NRC. Crucially, these correlations are derived simultaneously from a single unified model operating on a clinically-enrolled multi-center cohort, rather than from separate single-disease models applied to population-based registries. The progressive RBAG gradient from +0.8 years in healthy controls to +6.7 years in NRC is biologically coherent with documented pRNFL and mGCIPL thinning trajectories in Alzheimer’s and Parkinson’s disease. The cross-population validation by Zhu et al. (25), achieving a mean absolute error of 2.79 years across UK Biobank and three Chinese cohorts, further supports the cross-ethnic robustness of the retinal biological age paradigm in which RBAG participates.

Practically, RBAG offers a single continuous value that can be reported alongside the categorical output. Because it scales with severity across all three conditions, it could serve as a triage signal in screening settings, where a markedly elevated gap flags an eye for specialist referral regardless of disease category, and as a monitoring metric, where serial measurements track whether accelerated retinal aging is progressing or stabilizing. Its disease-agnostic nature is an advantage in primary care, where the specific systemic diagnosis may not yet be known. These uses remain to be confirmed prospectively, as the current evidence links RBAG to prevalent severity rather than future change.

### 4.2 Performance in the context of simultaneous multi-disease retinal AI

Prior deep learning studies in retinal disease detection have operated predominantly in a single-disease framework. A systematic review and meta-analysis by Ganji et al. (8), encompassing ten studies across Alzheimer’s and Parkinson’s disease, reported an overall pooled AUC of 0.73 (95% CI: 0.69–0.77; I^2^ = 78%), with OCT-based models achieving the highest diagnostic accuracy at AUC 0.762, reflecting the state of the art for unimodal single-disease approaches. A comprehensive review of AI-enhanced retinal imaging spanning cardiovascular, metabolic, cerebrovascular, and neurodegenerative conditions similarly documented that existing models largely operate on single modalities and single disease targets, identifying multimodal integration and simultaneous multi-disease stratification as the principal unmet needs in the field.

RetinalVNG-Net’s external AUC of 0.924 for NRC represents a substantial improvement over the meta-analytic benchmark, achieved simultaneously with AUC 0.963 for HR and 0.951 for DR within one multimodal inference. This suggests that simultaneous multi-disease risk stratification may not impose a substantial performance penalty over single-disease models when the fusion architecture is sufficiently expressive to disentangle disease-specific multimodal representations, though direct comparison with single-disease models trained on the same data would be needed to confirm this.

### 4.3 Generalizability and multi-device validation

Inadequate external validation across imaging devices represents perhaps the most commonly cited translational barrier in retinal AI. The Wang et al. (26) real-world deployment study demonstrated that RETFound-enhanced screening improved sensitivity and specificity by over 15% compared with commercial models in a Chinese community screening program spanning multiple camera types. RetinalVNG-Net’s validation-to-external AUC gap of only 0.013, achieved across Center C’s Canon CR-2 fundus camera and Heidelberg Spectralis OCT, neither appearing in training, confirms that CycleGAN-based domain normalization effectively mitigated inter-device variability. The inter-center AUC comparison within internal validation (Center A: 0.959, Center B: 0.954, *p* = 0.38) provides corroborating evidence that domain harmonization may have reduced device-specific variability, supporting further evaluation of deployment robustness across heterogeneous real-world imaging infrastructure. A central caveat is that all three centers were in one geographic region and the cohort was predominantly of East Asian ancestry. Retinal manifestations of systemic disease may differ across ethnic groups, and population-level differences in fundus pigmentation and in normative pRNFL and mGCIPL thickness ranges could shift both imaging features and the OCT-based component of the NRC definition. CycleGAN normalization reduced inter-device variability but cannot correct for genuine population-level differences. Performance on African, European, and South Asian populations therefore remains uncharacterized, and the device-agnostic RBAG claim should be read as provisional pending the multi-ethnic validation described in Section 4.4.2.

### 4.4 Limitations and future directions

#### 4.4.1 Research limitations

Several limitations of this study should be considered when interpreting the findings.

First, the retrospective design introduces potential selection bias, as subjects were identified from existing clinical databases rather than through prospective enrollment. While we applied rigorous inclusion and exclusion criteria, unmeasured confounding variables may influence model performance. Prospective validation studies with consecutive enrollment are needed to establish real-world performance characteristics.

Second, all three centers were located in the same geographic region (Northeast China), which limits generalizability to diverse ethnic populations and healthcare settings. The retinal manifestations of systemic diseases may vary across ethnic groups due to genetic, environmental, and dietary factors. External validation in multi-ethnic international cohorts, including populations of African, European, and South Asian descent, is essential before broader clinical deployment.

Third, the neurodegenerative-associated retinal changes (NRC) category combined Alzheimer’s disease and Parkinson’s disease into a single class. While both conditions share features of neuroretinal degeneration, they have distinct pathophysiological mechanisms and retinal signatures. This aggregation may obscure disease-specific patterns and limits the clinical utility for differential diagnosis. Future work should aim to develop multi-class discrimination among specific neurodegenerative conditions.

A directly related consideration concerns the NRC label itself, which combined a neurological diagnosis with an OCT-based thinning criterion while OCT data simultaneously served as a model input. This raises a potential for circularity between the label and the imaging features. The sensitivity analyses in Section 3.11 mitigate this concern, showing that discriminative performance persisted when the thickness sub-stream was removed (AUC 0.902) and when NRC was defined by neurological diagnosis alone (AUC 0.883). Nonetheless, some residual dependence between the label definition and the imaging features cannot be fully excluded, and the diagnosis-only formulation represents the more clinically meaningful target for future studies.

Fourth, the cross-sectional nature of this study precludes evaluation of prognostic performance. The RBAG biomarker’s ability to predict incident disease, disease progression, or treatment response remains to be established through longitudinal follow-up studies. The current findings demonstrate association with prevalent disease severity but not prediction of future outcomes.

Fifth, patients with coexisting target conditions were excluded to avoid label ambiguity. While this preserves diagnostic certainty for model training, it does not reflect real-world clinical populations where multimorbidity is common. The model’s performance in patients with multiple concurrent conditions remains unevaluated and represents an important direction for future investigation. Because comorbid cases were removed, the four classes were rendered mutually exclusive, which simplifies the discrimination task and likely yields an optimistic estimate of performance relative to unselected clinical populations, where hypertension, diabetes, and neurodegeneration frequently co-occur in the same patient. Reformulating the problem as multi-label classification, allowing a subject to carry more than one condition simultaneously, is therefore a necessary next step before clinical deployment.

Sixth, the training data were predominantly derived from patients of East Asian ancestry. Although the CycleGAN-based domain normalization successfully harmonized images across devices, it cannot address potential differences in disease manifestation across ethnicities. Validation in diverse populations is necessary to ensure equitable performance.

Seventh, the computational requirements of RetinalVNG-Net (approximately 105.5 million trainable parameters, 140 GPU-hours for complete experimentation) may limit deployment in resource-constrained settings. Model compression techniques, including knowledge distillation and quantization, should be explored to facilitate broader implementation. That said, the sub-second GPU inference time reported in Section 2.11 suggests the principal deployment constraint is hardware availability for the OCT branch rather than prediction latency itself.

Eighth, the study did not include OCT angiography, which provides direct visualization of the retinal microvasculature and may offer additional discriminatory power, particularly for early neurodegenerative detection where capillary dropout may precede structural thinning. Integration of OCT angiography represents a promising avenue for enhancing NRC detection.

Finally, the clinical utility and cost-effectiveness of this multimodal approach compared to standard care pathways have not been evaluated. Formal health-economic analyses and implementation science studies are needed to determine whether the incremental performance gain justifies the additional complexity and cost of multimodal imaging in routine practice.

#### 4.4.2 Future directions

Addressing these limitations, our ongoing work includes:

- Prospective validation in community-based screening programs across three additional centers in Southern China and Singapore, with consecutive enrollment to minimize selection bias
- Longitudinal follow-up of the current cohort to assess RBAG as a predictor of incident cardiovascular events (myocardial infarction, stroke) and neurodegenerative disease diagnosis over a 5-year horizon
- Development of a light-weight version of RetinalVNG-Net optimized for edge deployment using knowledge distillation and quantization, targeting a 5 × reduction in parameters with less than 2% performance degradation
- Incorporation of OCT angiography to evaluate its additive value for early NRC detection, particularly in patients with normal retinal thickness but evidence of microvascular dropout
- Multi-ethnic validation through collaboration with international consortia, including the UK Biobank, Singapore Epidemiology of Eye Diseases (SEED) study, and the African Descent and Glaucoma Evaluation Study (ADAGES)
- Health-economic modeling to estimate cost-effectiveness across different healthcare settings, including primary care screening programs, ophthalmology clinics, and neurology referral pathways

These investigations will help determine whether the promising technical performance of RetinalVNG-Net translates into meaningful clinical utility and improved patient outcomes across diverse global populations.

## 5 Conclusion

The retina encodes clinically meaningful signatures of systemic vascular and neurodegenerative pathology that conventional single-modality, single-disease AI frameworks have only partially exploited. RetinalVNG-Net demonstrates that integrating fundus photography, dual-stream OCT, and clinical metadata through cross-attention fusion enables simultaneous risk stratification of three major systemic disease categories with external macro-averaged AUC-ROC of 0.944. Generalizability was demonstrated across independent imaging platforms within a single geographic region, and broader generalization across diverse patient populations remains to be established. The RBAG biomarker extends this framework beyond categorical diagnosis, providing a continuous, exploratory index of accelerated retinal ageing that was independently associated with disease severity across all target conditions in this cohort. Its robustness across devices and populations requires further validation before it can be considered device-agnostic. Realizing the clinical potential of this approach will require prospective validation in globally representative populations and formal implementation science evaluation. The present findings provide evidence for the biological plausibility, technical feasibility, and cross-device generalizability of unified multimodal retinal AI, supporting further investigation as a potential platform for risk stratification of systemic disease-related retinal changes, with early, predictive detection remaining a goal for future longitudinal study.

## Data Availability

The original contributions presented in the study are included in the article/Supplementary material, further inquiries can be directed to the corresponding author.
