# Supplementary material for: Artificial intelligence-integrated multimodal retinal imaging for early detection and risk stratification of systemic vascular and neurodegenerative diseases
Source: Front Neurol. 2026 Aug 12;17:1885303. doi: 10.3389/fneur.2026.1885303 (PMC13506263; doi:10.3389/fneur.2026.1885303)
Supplement: Supplementary file 1 [file Table_1.DOCX]

**Supplementary Table S1**. Per-class external test AUC for metadata-only, imaging-only, and the full model

| **Class** | **Metadata-only (Random Forest)** | **Imaging-only (no metadata)** | **Full model** |
| --- | --- | --- | --- |
| HR | 0.842 | 0.951 | 0.963 |
| DR | 0.836 | 0.940 | 0.951 |
| NRC | 0.761 | 0.915 | 0.924 |
| HC | 0.794 | 0.927 | 0.938 |
| Macro | 0.812 | 0.929 | 0.944 |
